# Ultrasound‐guided percutaneous peritoneal dialysis catheter insertion using multifunctional bladder paracentesis trocar: A modified percutaneous PD catheter placement technique

**DOI:** 10.1111/sdi.12862

**Published:** 2020-03-11

**Authors:** Zhen Li, Hongyun Ding, Xue Liu, Jianbin Zhang

**Affiliations:** ^1^ Department of nephrology YongChuan Hospital ChongQing China; ^2^ Department of Medical Ultrasonics YongChuan Hospital ChongQing Medical University ChongQing China

**Keywords:** catheter leakage, catheter repositioning, catheter survival, multifunctional bladder paracentesis trocar, PD catheter, ultrasound guidance

## Abstract

**Background:**

To evaluate the efficacy and safety of ultrasound‐guided percutaneous peritoneal dialysis catheter insertion using multifunctional bladder paracentesis trocar.

**Methods:**

A retrospective review of 103 ESRD patients receiving percutaneous PD catheter insertion using a multifunctional bladder paracentesis trocar under ultrasound guidance at a single center between May 2016 and May 2018. Mechanical complications and catheter survival were evaluated over a 12‐month follow‐up.

**Result:**

Catheterization using this technique required only 10‐30 minutes from the beginning of local anesthesia to the end of skin suture at the puncture site (mean 18 ± 7 minutes) and an incision length of 2‐4 cm. Moreover, only four of 103 cases required catheter removal due to poor drainage within one month after surgery, with a success rate of 96.19%. Among failures, omentum wrapping was cause in two cases, catheter displacement in one case, and protein clot blockage in one case, while there were no instances of organ injury, severe hemorrhage, peritubular leakage, hernia, peritonitis, or exit infection within one month of PD catheter insertion. Catheter survival at 1 year was 92.2%.

**Conclusion:**

Percutaneous PD catheter insertion using a multifunctional bladder paracentesis trocar and ultrasound guidance is a feasible technique for ESRD patients.

## 1 INTRODUCTION

Accurate catheter insertion is a prerequisite for successful peritoneal dialysis (PD), and ease of insertion is predictive of PD success.^1^, ^2^ At present, the most commonly used methods for PD catheterization are open surgery, laparoscopic surgery, and percutaneous puncture based on the Seldinger technique.^3^, ^4^, ^5^ Of these, open surgery is currently the most common in clinical practice, as it is suitable for most patients, accurate, and reliable, and there are generally few complications. However, it is traumatic to patients and technically difficult, and thus must be completed by experienced surgeons.^6^ Laparoscopic surgery allows for precise localization of the catheter tip in the pelvic cavity under direct visualization, and requires only a small surgical incision that decreases surgery time and accelerates postoperative recovery. Also, simultaneous intra‐abdominal adhesiolysis can be done in cases with abdominal adhesion due to previous surgery.^7^ However, laparoscopic surgery requires general anesthesia and the creation of two or three holes in the abdominal wall by surgeons experienced in laparoscopic surgery, thus increasing the technical difficulty and limiting its applicability.

Alternatively, percutaneous puncture is a bedside operative technique conducted mainly by nephrologists based on Seldinger technology. It is a simple procedure with confirmed efficacy and so is attracting increasing attention from nephrologists.^8^ Further, percutaneous placement of PD catheters is performed under local anesthesia with minimal transcutaneous access, thereby facilitating rapid recovery.^9^ However, using the blind Seldinger technique, it is impossible to look directly into the pelvic cavity, and difficult to accurately place the dialysis catheter in the appropriate position based on feel. Thus, the catheter may be placed too deeply, stimulating the rectum and causing discomfort to the lower abdomen, or too superficially, increasing the risk of displacement and omentum wrapping. Therefore, the Seldinger technique is generally not suitable for obese patients or patients with a history of abdominal surgery. Further, the relatively expensive puncture components with avulsion sheath are not readily available in some small centers, that also hinders the wider application of this technique.

To enhance the technical ease and safety of PD catheter placement, we have improved the blind Seldinger technique by incorporating ultrasound guidance and the use of a multifunctional cystostomy paracentesis trocar for percutaneous puncture. The multifunctional cystostomy paracentesis trocar component has integrated functions of sharp‐headed trocar core puncture, blunt‐headed trocar core guidance, and semi‐ring outer sheath blunt dilation by pulling out the built‐in trocar core. The guidewire and catheter are placed through the semi‐ring sheath without the need for a separate dilator or the assistance of an avulsion sheath. This new technique can be easily performed by a nephrologist and is safe for PD patients. In this study, we report our experience with percutaneous PD catheter insertion using a multifunctional bladder paracentesis trocar and ultrasound guidance.

## 2 MATERIALS AND METHODS

### 2.1 Study sample and protocol

This is a retrospective analysis of all PD catheter (PDC) insertions using our new technique between May 2016 and May 2018, 105 PD catheters were placed in 103 ESRD patients who needed PD therapy at our center. Five of the 103 patients had a history of abdominal surgery (two patients with cesarean section and three with appendix surgery). Patient characteristics are summarized in Table 1.

**Table 1:** Baseline patient characteristics

Patients (n)	103
Catheters (n)	105
Female gender (n)	43
Mean age (y)	49.21 ± 14.15
Diabetic nephropathy [n(%)]	21 (20.4%）
Glomerulonephritis [n(%)]	60 (58.3%）
Hypertensive nephropathy [n(%)]	10 (9.7%)
Polycystic kidney disease [n(%)]	8 (7.8%)
Previous abdominal surgery [n(%)]	5 (4.8%)
Body mass index (BMI)	25.60 ± 2.51
Baseline eGFR	8.9 ± 3.7
Hemoglobin (g/dL)	9.1 ± 2.6
Albumin (g/dL)	3.7 ± 1.2
Blood urea nitrogen (mg/dL)	78.3 ± 13.4
Creatinine (mg/dL)	9.9 ± 3.0
Previous abdominal surgery [n(%)]	5 (4.8%)

**Figure 1:**
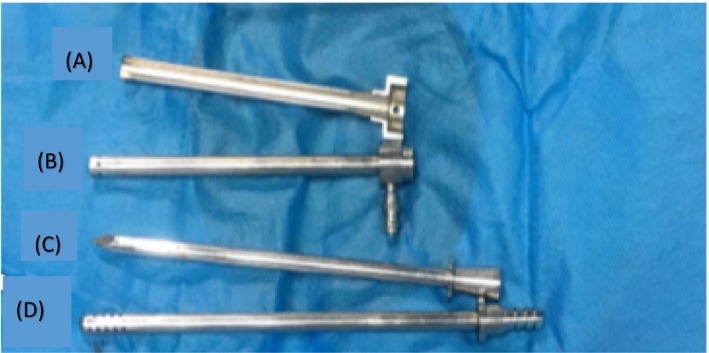
Details of the multifunctional bladder paracentesis trocar. The multifunctional cystostomy paracentesis trocar was an 18F stainless steel kit consisting of a semi‐ring outer sheath (A), an inner trocar sheath (B), a sharp‐headed trocar core (C), a blunt‐headed trocar core (D) [Color figure can be viewed at wileyonlinelibrary.com]

The primary endpoint of this study was a functional catheter one month postinsertion (defined as technical success). Potential early complications considered for analysis were bowel perforation, hemorrhage in the rectus muscle or pelvic cavity, peritonitis, pericatheter leakage, and poor drainage. The secondary endpoint was 1‐year technical survival, that was analyzed separately with regard to the insertion technique. The PD catheters used consisted of a double‐cuff Tenckhoff straight tube (Baxter) with an overall length of 41 cm and a diameter of 0.5 cm, in which the first polyester sheath was 16 cm from the catheter tip (Quinton Instrument Company, Seattle, WA). The multifunctional cystostomy paracentesis trocar was an 18F stainless steel kit produced by Dongai Medical Devices (Zibo, Shandong Province, China) consisting of an semi‐ring outer sheath, an inner trocar sheath, a sharp‐headed trocar core and a blunt‐headed trocar core (Figure 1), which is originally designed as a puncture kit for a bladder ostomy for patients with blocked urethra and inability to urinate normally.

This study was conducted with the approval of the institutional ethics committee. Prior to the procedure, we obtained informed consent from all patients to review their documents for research purposes.

### 2.2 Procedure details

**Figure 2:**
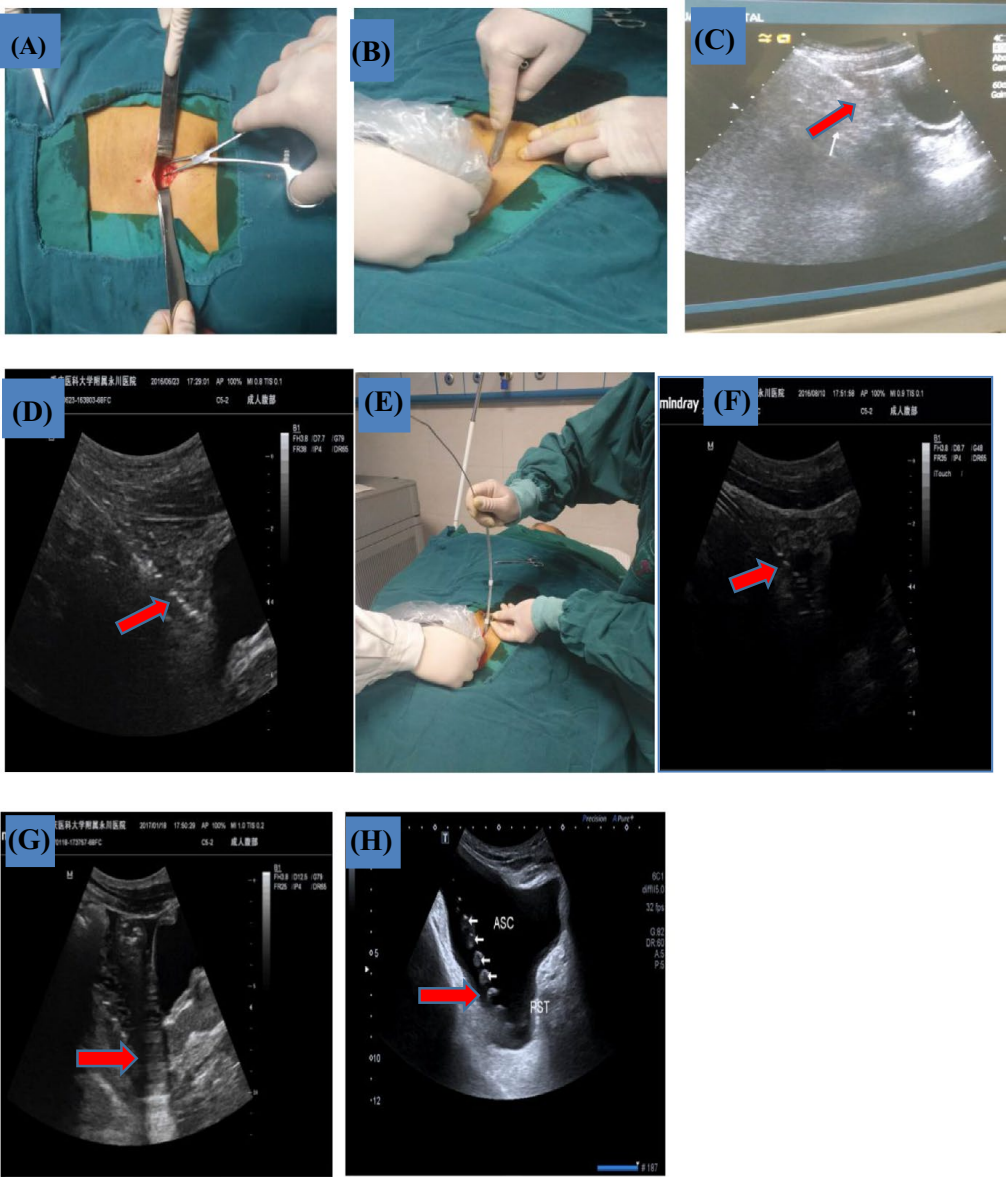
(A) Subcutaneous adipose tissue was cut and separated to reach the rectus abdominis muscle. (B and C) Under ultrasound, the multifunctional bladder paracentesis trocar was slowly rotated obliquely and pierced into the abdominal cavity (B: operator's view, C: ultrasonic display screen view). (D) After piercing into the abdominal cavity, the sharp‐headed trocar core was replaced by the blunt‐headed trocar core, and the blunt‐headed trocar core continued to move forward toward the vesicorectal fossa (or rectouterine pouch) under ultrasound guidance. (E and F) The trocar core was pulled out and then inserted into the peritoneal dialysis catheter through the outer sheath of the cannula trocar, and the tip of the catheter was confirmed by ultrasound to reach the Douglas pouch (E: operator's view, F: ultrasonic display screen view). (G) Liquid entering the vesicorectal pouch (or rectouterine pouch) concomitant with catheter injection of normal saline was observed under ultrasound to confirm correct catheter tip placement. (H) Location of the PD catheter tip after intraperitoneal injection of PD solution [Color figure can be viewed at wileyonlinelibrary.com]

All PD catheter insertions were performed by the same nephrologist in a procedure room designated by the nephrology ward. With the patient in the supine position, along the Parastolic median line of the abdominal wall and upward 10–12 cm from the superior margin of the symphysis pubis was selected as the puncture site. The location of the catheter in the pelvis and position of the deep cuff and the desired exit site of the PD catheter was marked preoperatively. Local infiltration anesthesia was performed with 2% lidocaine injection. A 2‐4 cm linear incision centered at the puncture point was made, followed by blunt separation of subcutaneous tissue to expose the anterior sheath of the rectus abdominis [Figure 2a]. A 0.5‐cm incision was made in the sheath and it was punctured at the center with a 50 ml syringe. Physiological saline (about 500 ml in total) was injected into the abdominal cavity to push the greater omentum and parietal peritoneum away. At the same time, an ultrasonic probe inserted into a disposable sterile endoscope sleeve was placed on the abdominal wall of the puncture site, and the 18F multifunctional cystostomy trocar was rotated slowly into the abdominal cavity at an oblique angle of 60 degrees [Figure 2B,C] under ultrasound monitoring. After breaking through the abdominal wall, the sharp‐headed trocar core was replaced by the blunt‐headed trocar core and insertion continued in the direction of the vesicorectal fossa (or rectouterine fossa) under color ultrasound guidance [Figure 2d]. After reaching the target area, the blunt‐head trocar core was pulled out. The outer sheath of the cannula trocar was inserted into the PD catheter with a guidewire and pulled out from the outer sheath of the paracentesis trocar. Under ultrasound, it was confirmed that the end of the catheter that reached the Douglas pouch [Figure 2e,f], was close to the proximal cuffs, blocked the rectus abdominis puncture hole, and was buried in the rectus abdominis muscle. The guidewire was then pulled out. To test for proper tip insertion, normal saline was injected through the catheter to confirm bubbling in the vesicorectal pouch (or rectouterine pouch) [Figure 2g] and linear drainage of fluid. A stitch at the lower end of the rectus abdominis anterior sheath puncture was used to fix the catheter cuff flat onto the rectus abdominis and establish a subcutaneous tunnel for exit of the external segment. Normal saline was then injected two or three times into the abdominal cavity (500 mL per injection) to confirm smooth drainage, no liquid leakage, and no intraluminal hemorrhage. Finally, the subcutaneous tissue and skin were sutured. Considering that the small incision length and surgical time from the beginning of local anesthesia to the end of skin suture was about 10‐30 minutes, patients were not given prophylactic antibiotics before surgery.

### 2.3 Postoperative treatment

After surgery, low‐dose heparin saline was injected into the abdomen, and 1.5% peritoneal dialysis solution was used to wash the abdominal cavity every day (four times, 500 mL each time). Routine Continuous ambulatory peritoneal dialysis (CAPD) treatment was started from 7 days after catheterization. The surgical dressing was changed every three days after surgery until the stitches were removed, and routine maintenance of the exit site was performed daily. During hospitalization, patients were provided with relevant knowledge for home PD treatment, including the early identification and treatment of peritonitis and exit site infection.

### 2.4 Indications for catheter removal

Conditions requiring catheter removal included difficult drainage, failure of manual reduction due to omentum wrapping and displacement, refractory peritonitis, recurrent peritonitis, fungal peritonitis, refractory exit and tunnel infection, reproducible peritonitis, mycobacterial peritonitis, and multiple intestinal bacterial infectious peritonitis. Catheters were reinserted following relief of peritonitis.

### 2.5 Data collection and definition

Data recorded during the catheterization procedure included incision length, catheterization time, and intraoperative complications. Conditions recorded after catheterization included pain, location of the catheter as confirmed by abdominal plain film, drainage obstruction, the color of peritoneal dialysis fluid, and the time from insertion to PD initiation. Complications evaluated regularly within the first month and at subsequent monthly follow‐ups included infection at the catheter exit, tunnel infection, catheter displacement, peritubular leakage, poor initial drainage, and peritonitis. All mechanical complications and catheter survival were evaluated over a 12‐month follow‐up.

Infection at the exit site was defined by the presence of purulent secretion with or without skin redness. When the egress tunnel became red and swollen or tender, ultrasonic examination was conducted to evaluate potential infection scope and curative effects during treatment. Peritonitis was defined by the presence of at least two of the following conditions: (a) abdominal pain and turbid peritoneal dialysis fluid with or without fever, (b) white blood cell count in PD effluent >100 × 106/L and proportion of neutrophils >50%, and (c) growth of pathogenic microorganisms from cultivation of the effluent. Catheter leakage and peritubular leakage were defined as dialysate drainage from the exit site or main wound. Abdominal plain film examination was performed in case of suspected catheter displacement. Poor initial drainage was defined as drainage of <50% of the fill volume.

### 2.6 Statistical analysis

Continuous variables are expressed as mean ± standard deviation and categorical data as number (%). Categorical variables were compared using chi‐square test or Fisher exact test. Catheter survival rate was calculated from the day of insertion to the day of removal. A *P* < .05 was considered statistically significant. Statistical analysis was performed using SPSS version 23.

## 3 RESULT

Procedural and postprocedural details are summarized in Table 2. In total, 105 catheters were inserted by ultrasound‐guided percutaneous puncture using a multifunctional bladder paracentesis trocar. Of these, 103 were first insertions and two were second insertions after the first catheter removal of which one exhibited omental wrapping within one month postinsertion, one drainage disturbance caused by catheter tip displacement just 13 months postinsertion, both patients have a functioning catheter after replacement follow‐up 1 year.

**Table 2:** Operative characteristics and early^a^ complications

Total patients	105
Operation time (min)	10‐30
length of incision(cm)	2‐4
Postoperative analgesic needs	0
Bowel perforation	0
Hemorrhage in rectus muscle or pelvic cavity	0
Poor initial drainage [n (%)]	5 (4.76%）
Early peritonitis [n (%)]	0
Tunnel infection [n (%)]	0
Catheter migration [n (%)]	1 (0.95%)
leakage	0
Mortality N (%)	0
Primary failure [n (%)]	4 (3.81%）
Success rate [n (%)]	101 (96.19%）

^a^ Within one month of PDC insertion.

Incision length was 3.0 ± 0.8 cm and insertion time was 20.7 ± 5.7 minutes (from 10 to 30 minutes). No patient required analgesics after catheterization. Abdominal plain film ultrasound revealed that catheter location was normal on the day following insertion, and there were no cases of major organ injury, severe abdominal hemorrhage, catheter displacement, peritubular leakage, incision hernia, early peritonitis, exit infection, or tunnel infection. Drainage obstruction occurred in five cases within one month, including two cases with catheter tip displacement. Of these, one case returned to normal after manual reduction, while the other cases required surgical reduction. Omentum wrapping and blockage occurred in three cases, all requiring surgical reduction or catheter replacement.

Table 3 summarizes data related to the primary endpoint. Clinical success was defined as a functional catheter for at least one month after PD catheter insertion. Of 105 catheters inserted, 101 insertions (96.2%) were clinically successful. Of the four catheters requiring surgical correction, three occurred in patients without a history of previous abdominal surgery and one in a patient with a history of previous abdominal surgery. These relative incidences (three of 100 patients with no history of surgery and one of five with a history of surgery) suggest that prior surgery may impede success, an issue warranting additional study in a larger cohort.

**Table 3:** Clinical success of PD catheter insertion

Patients	Number	Percentage
Total (N)	105	
Functioning catheter	101	96.19%
Needing revision	4	3.81%
Previous abdominal operation (N)	5	
(a) Functioning catheter	4	80%
(b) Needing revision	1	20%
Virgin abdomen (N)	100	
(a) Functioning catheter	97	97%
(b) Needing revision	3	3%

**Figure 3:**
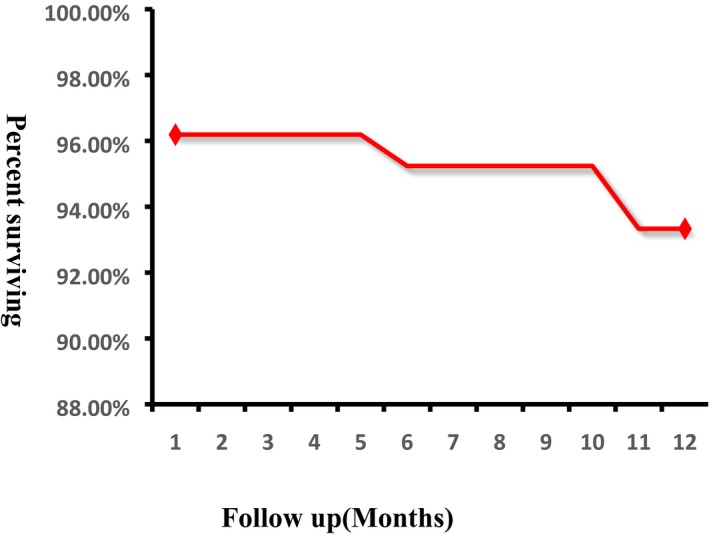
Technical survival of peritoneal dialysis (PD) catheters inserted by Ultrasound guided percutaneous technique using multifunctional bladder paracentesis trocar [Color figure can be viewed at wileyonlinelibrary.com]

Table 4 summarizes the causes of catheterization failure. Of the four patients with catheterization failure, two exhibited peritoneal wrapping, one drainage disturbance caused by catheter tip displacement, and the other protein blockage due to failure of urokinase sealing. One failure case received catheter removal and replacement, two cases underwent surgical reduction, and one case catheter removal and transfer to hemodialysis treatment. The catheter survival curve is shown in Figure 3. The one‐year survival rate was 92.20%.

**Table 4:** Reasons for failure of the PD catheter

Reasons	Number	Percentage
Total (N)	4	
Omental wrapping	2	50%
Catheter migration, N (%)	1	25%
Others, N (%)	1	25%

## 4 DISCUSSION

Accurate and stable catheter insertion is necessary for successful peritoneal dialysis. In this study, the rate of successful catheter insertion by nephrologists was significantly higher than achieved by surgeons using open or laparoscopic procedures.^3^, ^4^, ^10^ Further, our technique can be completed in a more timely manner due to its simplicity as a bedside procedure, while surgical laparotomy may be delayed by operating room and staff availability, that places additional stress on the patient. Further, the lack of interest in catheterization by some central surgical teams is another limiting factor in the initiation of PD.

Bedside percutaneous puncture based on Seldinger technology has attracted the attention of nephrologists as several small‐sample studies have demonstrated efficacy and safety comparable or superior to traditional insertion by surgery.^11^ However, patients with laparotomy history, severe or recurrent peritonitis, morbid obesity, anatomical abnormalities, or increased risk of hemorrhage may be more suited to surgical insertion. Moreover, the conventional Seldinger technique uses a blind puncture process, so it is difficult to place the catheter accurately and there is risk of organ injury and hemorrhage. Some studies have even found no advantages of percutaneous catheterization over traditional open surgery or laparoscopic surgery regarding catheter‐related complications and catheter survival duration,^12^, ^13^, ^14^, ^15^ suggesting the need for further improvement.

We improved the blind Seldinger technique by incorporating ultrasound guidance and use of a multifunctional bladder paracentesis trocar with integrated puncture, guidance, and blunt dilator (semi‐ring outer sheath) functions. After pulling out the built‐in trocar core, the guidewire and catheter are placed through the semi‐ring sheath using the blunt dilator function without the need for a separate dilator or the assistance of an avulsion sheath. Also, with the help of color ultrasound guidance, it is possible to avoid blind puncture and effectively prevent organ injury, severe bleeding, and other complications. Indeed, the surgical incision was only about 2‐4 cm and surgical period was 10‐30 minutes, that obviated the need for analgesia after insertion. The success rate was high (96%), as only five of 105 cases exhibited early drainage disturbance (within 1 month after surgery), of which one case recovered after manual reduction. Thus, only four of 105 cases required surgical reduction. Medani et al reported a 90% success rate, defined as maintenance of normal catheter function for 2‐4 weeks, using the blind Seldinger technique in patients receiving catheterization for the first time without a history of abdominal surgery, similar to that of a parallel surgical placement group.^16^ However, van Laanen et al reported that only 77% of patients maintained normal catheter function for 2 weeks after surgical insertion (failure rate of 23%),^11^ and several other randomized control trials have also reported relatively high (16% to 30%) failure rates for catheterization by surgical laparotomy.^12^, ^13^, ^14^ Thus, all the previous techniques show substantially greater failure rates (up to 30%) than ultrasound‐guided percutaneous peritoneal dialysis catheter placement using a multifunctional bladder paracentesis trocar (around 4% failure).

The most common complications affecting the success of PD catheter placement include omental wrapping, malpositioning of the catheter between bowels, and catheter migration.^15^ Omental wrapping was also the most common complication of our technique. Although the sample was small and requires confirmation, the failure rate (three of 105 cases, 2.86%) is lower than reported for surgical laparotomy and the blind Seldinger percutaneous puncture technique, likely due to enhanced accuracy of placement in the Douglas fossa under ultrasound guidance. If elastic resistance is encountered during advancement of a blunt‐headed trocar with guidance function, or if a floating omentum and blunt‐headed trocar moving into the pelvic cavity are observed by color ultrasound, the trocar can be pulled out slightly to adjust the angle and avoid the omentum, and then pushed forward again into the Douglas fossa, so as to effectively prevent the catheter from being wrapped or displaced by the omentum due to shallow placement. This step proved essential for our high success rate compared to surgical methods and the blind Seldinger technique.

Percutaneous placement as a "blind" technique carries the risk of inadvertent puncture of the abdominal viscera, and although the reported incidences of organ injury and severe bleeding are low, these concerns have limited the widespread use of this technique. We used real‐time ultrasound guidance to avoid blind puncture, and so effectively eliminated this risk. So far, none of our patients have experienced organ injury or severe bleeding. In previous studies, the incidence of postoperative catheter leakage was the most frequent mechanical complication in the percutaneous puncture group, with incidence ranging from 2.6% to 22%, higher than among surgical placement groups.^14^, ^16^, ^17^, ^18^ Using our technique, there was no case of catheter leakage, likely due in part to the small incision hole and blockade of the rectus abdominis muscle puncture hole using the proximal cuff after blunt separation. Further, these steps reduced unnecessary injury and so contributed to rapid recovery.

Infection is the main cause for catheter removal.^19^ Although none of the patients in our study received antibiotics to prevent peritonitis, there were no such cases. In contrast, retrospective studies have reported incidences of 20.2% using blind percutaneous insertion and 27.1% using laparoscopy.^20^, ^21^ In some reports, incision and tunnel infection have occurred regardless of technique (surgical laparotomy, percutaneous puncture, or laparoscopic catheterization).^5^, ^20^, ^22^ We believe that our new PD catheterization method has inherent advantages in this regard.

The survival duration of catheter insertion is the most important factor for PD implementation. A randomized trial by van Laanen et al reported a 1‐year survival rate of only 70% in the laparotomy group and 60% in the laparoscopic group,^7^ while three prospective randomized trials reported survival rates of 67%─84%,^23^, ^24^, ^25^ substantially lower than the 92.2% reported in this study. Thus, percutaneous PD catheter insertion using a multifunctional bladder paracentesis trocar and ultrasound guidance may facilitate more successful PD.

This study has several limitations. First, we did not directly compare this technique to other catheter placement methods at our center, so it remains uncertain whether this technique is superior in general or for specific patients. Second, this was a single‐center study with a relatively small number of patients and a short follow‐up period. Based on these results, a large‐scale multicenter randomized study comparing insertion techniques is warranted. Third, it is not possible to completely rule out selection bias. For instance, the vast majority of patients had no previous history of abdominal surgery. Fourth, the technique requires further improvement. For instance, it is impossible to conduct adhesion lysis and omentectomy with the current version of this technique.
